# Conduction Abnormalities After Transcatheter Aortic Valve Replacement: Comprehensive Review of Current Literature, Guidelines, and Clinical Practices

**DOI:** 10.31083/RCM48122

**Published:** 2026-04-09

**Authors:** Kamran Namjouyan, Matthew Yeckley, Neria Bitton, Madeleine Marcus, Christopher Eaton, Aravdeep Jhand

**Affiliations:** ^1^Heart Institute, Geisinger Medical Center, Danville, PA 17821, USA; ^2^Medicine Institute, Geisinger Medical Center, Danville, PA 17821, USA; ^3^Department of Structural and Interventional Cardiology, Geisinger Medical Center, Danville, PA 17821, USA

**Keywords:** transcatheter aortic valve replacement, aortic valve stenosis (therapy), heart conduction system (injuries), atrioventricular block (etiology), bundle-branch block (complications), pacemaker, artificial (adverse effects), risk assessment, prosthesis implantation (methods)

## Abstract

Transcatheter aortic valve replacement (TAVR) offers a minimally invasive alternative to traditional surgical aortic valve replacement (SAVR) for the treatment of severe aortic stenosis. Notably, TAVR was once reserved for patients at high surgical risk but is now a viable option even for those at low surgical risk. Despite the widespread adoption and favorable outcomes of TAVR, this technique presents several challenges, including conduction disturbances such as new-onset left bundle branch block (LBBB) and high-grade atrioventricular (AV) block, which may require permanent pacemaker (PPM) implantation. These complications arise from the close anatomical relationship between the aortic valve and the cardiac conduction system and are influenced by factors such as valve design, implantation depth, and individual anatomical variations. This review aims to explore the structural and physiological intricacies of the aortic valve and conduction system. Additionally, this review explores pre-procedural risk stratification, monitoring protocols, and emerging strategies to mitigate these complications and enhance procedural safety and long-term patient outcomes.

## 1. Introduction

Transcatheter aortic valve replacement (TAVR) is a minimally invasive procedure to treat severe and symptomatic aortic stenosis [1]. Aortic stenosis can develop due to various etiologies, including age-related calcification, congenital valve abnormalities (e.g., bicuspid aortic valve (BAV)), or rheumatic heart disease [2, 3]. To classify aortic stenosis as severe, both valve anatomy and hemodynamics must be considered. Severe aortic stenosis is diagnosed when the aortic velocity is ≥4.0 m/s, the mean transaortic gradient is ≥40 mmHg, and the aortic valve area is ≤1.0 cm^2^, with an indexed valve area of ≤0.6 cm^2^/m^2^ [1, 2]. According to a 2021 review published in the Journal of the American Medical Association (JAMA) on transcatheter treatment of valvular heart disease, approximately 78,000 TAVR procedures are performed annually. Notably, in 2019, TAVR surpassed surgical aortic valve replacement (SAVR), with 77,991 procedures compared to 57,626 [4]. TAVR was initially approved for patients deemed intermediate- to high-risk or inoperable for conventional open-heart surgery. However, it is now considered a viable first-line treatment for low-risk patients, although an individualized heart team approach remains essential for all cases [1, 4].

Multiple clinical trials have demonstrated that TAVR is noninferior or even superior to SAVR in certain populations [5, 6, 7]. However, SAVR may be preferred in patients with a thoracic aortic aneurysm requiring repair, severe coronary artery disease necessitating surgical revascularization, or significant left ventricular outflow tract (LVOT) calcification (which increases the risk of annular rupture during TAVR). Conversely, TAVR may be favored in patients with morbid obesity, severe pulmonary, hepatic, or renal disease, or those with prior chest irradiation [4].

The decision to undergo TAVR involves shared decision-making between the patient and a multidisciplinary heart team. Pre-procedural evaluation includes transthoracic echocardiography (TTE), coronary artery assessment, TAVR-specific computed tomography (CT), and dental examinations [1, 8]. The procedure typically involves transfemoral or carotid delivery of a prosthetic aortic valve, which is advanced over a guidewire and deployed across the native aortic valve [1, 4, 9]. Deployed prosthetic valve may be balloon-expandable or self-expanding, often performed during rapid ventricular pacing to reduce cardiac output. The placement of the valve is confirmed via fluoroscopy and echocardiography. Most procedures are conducted under monitored anesthesia, and patients are usually discharged within 1–3 days [4].

Despite its minimally invasive nature, TAVR carries risks such as stroke, paravalvular leak, myocardial infarction, and bleeding [8, 10, 11]. One particularly significant complication is conduction disturbances [10, 11]. These disturbances may include new persistent left bundle branch block (LBBB) or disturbances requiring permanent pacemaker implantation, which have been associated with increased heart failure hospitalizations and all-cause mortality at one year [12]. While some conduction disturbances resolve within 30 days, others persist and contribute to morbidity and mortality [13].

Latest technological advancements have reduced complications like paravalvular leak, but conduction disturbances remain prevalent. These are believed to result from mechanical trauma or compression and the depth of valve implantation. Newer-generation self-expanding valves and deeper implantation are associated with higher risk, although the ACURATE Neo valve has not shown increased risk with deep placement [14, 15]. Notably, the clinical presentations of aortic stenosis and bradyarrhythmia, such as fatigue, lightheadedness, and syncope, can overlap. Although routine cardiac monitoring prior to TAVR is not standardized, undiagnosed bradyarrhythmia may contribute to post-procedural conduction disturbances [10].

This review aims to explore the anatomy and physiology of the aortic valve and its anatomical variations, mechanisms of injury during TAVR, and the types of conduction disturbances that may occur. It will examine the incidence and clinical implications of conduction disturbances by valve type and generation, summarize pre-procedural risk factors, predictors, and monitoring guidelines, and discuss the role of permanent pacemaker (PPM) implantation and future directions for the TAVR procedure. A comprehensive literature search was conducted using institutional databases, including PubMed, Scopus, Embase, and Web of Science, focusing on peer-reviewed studies published between 2010 and 2025. Search terms included “transcatheter aortic valve replacement”, “aortic stenosis”, “conduction disturbances”, “left bundle branch block”, “atrioventricular block”, “pacemaker implantation”, “valve implantation depth”, and “procedural complications”. Studies were selected based on relevance to TAVR-related conduction injury, procedural mechanisms, valve-specific outcomes, and peri-procedural risk stratification. This review was conducted as a narrative, non-systematic review. Studies were prioritized based on clinical relevance, methodological rigor, sample size, and applicability to contemporary TAVR practice. When conflicting evidence was identified, greater weight was given to large registries, randomized trials, meta-analyses, and guideline statements, while single-center observational studies were used to provide mechanistic insight and hypothesis-generating data. Formal risk-of-bias tools and quantitative evidence weighting were not applied. Data extraction focused on reported incidence, predictors, valve-specific outcomes, and clinical implications of conduction disturbances following TAVR.

## 2. Anatomy and Pathophysiology of the Conduction System

**Fig. 1.**
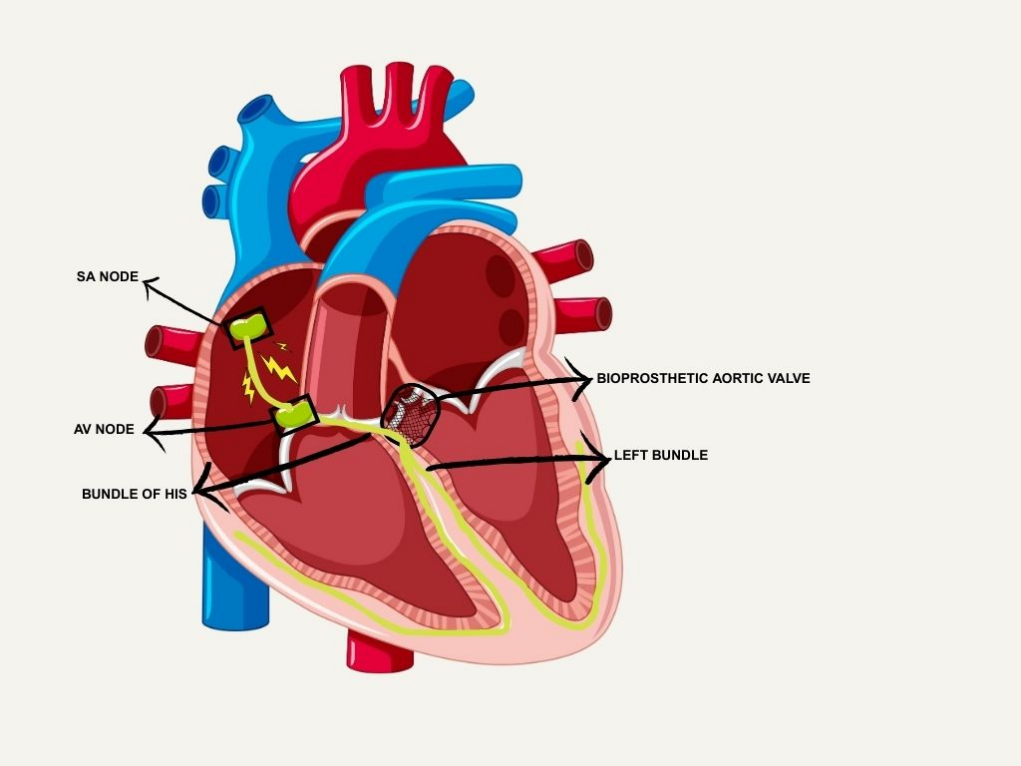
**Anatomy of the conduction system and its close proximity to the aortic valve**. SA, sinoatrial; AV, atrioventricular. Figure created with Canva.

The anatomical proximity of the native conduction system to the aortic valve complex underlies the most common conduction-related complications associated with TAVR. During valve manipulation and expansion, impingement on adjacent conduction tissue can result in permanent injury. The American College of Cardiology (ACC) highlights the risk of compressing the membranous septum (MS), in particular its most inferior portion, which contains the His bundle as it courses from the atrioventricular (AV) node to the ventricular conduction system [10, 11]. Fig. 1 illustrates the close proximity of the AV node, His bundle, and left bundle branch to the aortic valve annulus and LVOT, demonstrating the conduction system’s vulnerability during TAVR.

The mechanism of injury is typically multifactorial, which involves mechanical trauma, edema, ischemia, and occasionally hematoma formation. The 2020 ACC Expert Consensus notes that direct injury contributes to a 10%–15% incidence of complete heart block requiring permanent pacemaker (PPM) implantation [10]. Valve deployment may compromise microvascular supply, which can lead to ischemia of the AV node; one of the most common causes of high-grade AV block [10, 11]. Edema resulting from procedural manipulation may also transiently disrupt conduction.

Several anatomical and procedural factors increase susceptibility to conduction compromise. These include a short MS, aortic annular calcification, and a narrow LVOT [12, 13]. A shorter MS brings the aortic annulus closer to the conduction system and is frequently observed in patients with BAVs. While TTE often lacks sufficient resolution to assess septal length, TEE, cardiac CT, and cardiac magnetic resonance imaging (MRI) may provide more accurate evaluation [13]. Multidetector cardiac CT has emerged as the preferred pre-procedural imaging modality due to its great spatial resolution and reproducibility. Several CT-based analyses have demonstrated that a short membranous septal length (as ≤6–7 mm) is strongly associated with increased risk of new-onset LBBB and PPM following TAVR. Membranous septal lengths ≥8 mm appear to show relative protection, in particular when implantation depth is maintained above the inferior margin of the septum. Therefore, implantation extending beyond the measured septal length substantially increases the likelihood of His bundle or proximal left bundle branch compression. While TTE lacks sufficient resolution for reliable septal measurement, CT allows simultaneous assessment of membranous septal length, LVOT diameter, annular geometry, and prosthesis–LVOT interaction [14, 15, 16]. Additionally, variations in the trajectory of the AV bundle within the septum can influence risk. In some cases, the AV bundle runs superficially along the MS, making it more susceptible to injury from minimal trauma [17].

Calcification of the aortic annulus, especially beneath the non-coronary cusp, can serve as a focal point for increased compression [10, 18]. The ACC also emphasizes the importance of LVOT size in risk stratification. A narrow LVOT or a high prosthesis-to-LVOT ratio inherently increases the risk of conduction system compression, regardless of valve type [10]. Moreover, asymmetric calcium deposition may alter radial force vectors by resulting in localized stress concentration and disproportionate injury to conduction tissue. This explains the conduction disturbances that may occur even in cases with modest overall annular calcification. Incorporating calcium distribution patterns into pre-procedural CT assessment may therefore enhance individualized risk prediction and procedural planning [19].

## 3. Types of Conduction Disease 

### 3.1 Left Bundle Branch Block 

TAVR has the potential to cause various forms of conduction disturbances. In a physiologically normal heart, electrical conduction begins in the sinoatrial (SA) node and progresses sequentially through the AV node, His bundle, bundle branches, fascicles, Purkinje fibers, and ultimately to the ventricular myocardium [20]. The most frequently observed conduction disturbance following TAVR is new-onset LBBB, which has been reported in 19%–55% of patients post-procedure [20]. The diagnostic criteria for complete LBBB on electrocardiogram (ECG) in adults include a QRS duration >120 ms, broad notched or slurred R waves in leads I, aVL, V5, and V6, absence of Q waves in leads I, V5, and V6 (with possible Q waves in aVL), R peak time >60 ms in leads V5 and V6, and ST-T wave discordance relative to the QRS direction. Incomplete LBBB is defined similarly, but with a QRS duration between 110–119 ms, possible left ventricular hypertrophy pattern, R peak time >60 ms in leads V4–V6, and absence of Q waves in leads I, V5, and V6 [20].

While isolated LBBB or incomplete LBBB rarely causes overt physical symptoms, persistent LBBB can lead to long-term complications. Its incidence varies depending on valve type and generation. According to a meta-analysis and systematic review by Alzu’bi *et al*. [21], larger valves (e.g., 29 mm) are associated with an increased risk of LBBB (relative risk [RR] 1.59). A study by Isogai *et al*. [22] found that newer-generation valves, such as the SAPIEN 3, have a reduced but still notable risk of LBBB, potentially due to improvements in technique or valve design.

Despite procedural advancements, LBBB remains a significant concern, particularly with valves known to have higher associated rates. Alzu’bi *et al*. [21] reported that the self-expanding CoreValve had the highest incidence of LBBB (26%–30%), with a RR of 2.25. Similar findings were noted by Auffret *et al*. [23] in a review published in Circulation, where the incidence of new-onset LBBB with the CoreValve ranged from 18%–65%. In contrast, balloon-expandable valves such as the Edwards SAPIEN XT and SAPIEN 3 showed lower LBBB incidence, ranging from 4%–30% [23]. In two separate analyses of the PARTNER II trial by Nazif *et al*. [24, 25], the incidence of new LBBB was reported to be 3%–30% for the SAPIEN XT and SAPIEN 3 valves.

New-onset LBBB may appear immediately after valve deployment or up to one-year post-procedure, though it most commonly occurs peri-procedurally [21, 23]. While LBBB may resolve spontaneously within 24–48 hours, especially with the SAPIEN 3 valve, persistent LBBB beyond 24 hours is associated with a 30.6% risk of developing high-grade AV block within 12 months, according to a prospective multicenter study published in Heart Rhythm by Massoullié *et al*. [26].

### 3.2 High-Degree Atrioventricular Block 

Another conduction disturbance that can result from the TAVR procedure is high-degree atrioventricular block (HAVB). On ECG, HAVB is defined as two or more consecutive non-conducted P waves occurring at a constant physiological rate with some evidence of normal conduction elsewhere [20]. This type of block can lead to significant bradycardia and symptoms such as lightheadedness or syncope. HAVB is typically less responsive to pharmacologic interventions like atropine and has the potential to progress to complete heart block [20].

According to the ACC, American Heart Association (AHA), and Heart Rhythm Society (HRS), the overall incidence of high-grade AV conduction disturbances following TAVR is approximately 10% [10, 20]. However, studies such as Rao *et al*. [27] (a prospective observational study from the CONDUCT-TAVI trial), El-Sabawi *et al*. [28] (a single-center retrospective study), and Reiter *et al*. [29] (a single-center, prospective, nonrandomized cohort trial) report incidence rates as high as 21%, with most cases occurring within the first 48 hours post-procedure. Importantly, late-onset HAVB is defined as occurring more than two days after the procedure and has been observed in 8%–12% of cases. This highlights the need for close clinical monitoring, especially in patients with preexisting right bundle branch block (RBBB), who are at increased risk [26, 29, 30]. According to the ACC, the development of sustained or recurrent HAVB often necessitates PPM implantation. Furthermore, new persistent LBBB and conduction disturbances requiring PPM placement after TAVR have been associated with increased rates of heart failure hospitalization and all-cause mortality at one year [31].

### 3.3 Right Bundle Branch Block 

An additional, though relatively rare, conduction disturbance that can develop following the TAVR procedure is RBBB. While the 2020 ACC guidelines do not specify the exact incidence of new-onset RBBB post-TAVR, data from a large-scale registry at the Mayo Clinic and a single-center retrospective observational study by Kikuchi *et al*. [32] published in Journal of the American Heart Association suggest an incidence of approximately 4%–5% [10, 33].

RBBB is defined on an ECG by a QRS duration ≥120 ms, an rsR^′^ or rSR^′^ pattern in leads V1–V2, and a broad S wave in leads I and V6. Incomplete RBBB presents with similar QRS morphology but with a QRS duration between 110–119 ms [20]. RBBB results in delayed right ventricular activation, as the electrical impulse must travel through the left bundle branch and then across the interventricular septum. Like isolated LBBB, isolated RBBB typically does not produce specific physical symptoms [20].

In the study by Kikuchi *et al*. [32] involving 407 patients undergoing TAVR, the rate of PPM placement was higher in patients who developed new-onset RBBB compared to those with new-onset LBBB or no bundle branch block [29]. Additionally, the ACC notes that preexisting RBBB is a strong independent predictor of post-TAVR conduction disturbances, with rates of high-grade AV block reaching as high as 24% in this population [10]. These findings suggest that although new-onset RBBB is relatively uncommon, it represents a high-risk complication that may necessitate PPM implantation.

Michowitz *et al*. [34] evaluated over 7700 TAVR patients and reported a 0.5% (5.3/1000) incidence of new-onset RBBB, nearly half of whom required permanent pacemaker implantation, primarily within the first week post-procedure. A PR interval ≥230 ms or ΔPR ≥24 ms were robust predictor for pacing need. These findings reinforce the importance of prolonged telemetry (up to 7 days), mainly in patients presenting post-TAVR PR prolongation, to identify high-grade conduction disturbances requiring intervention [34].

### 3.4 Other Arrhythmias

Atrial fibrillation (AF), which is a common arrhythmia among the severe aortic stenosis population, can also develop or be unmasked following the TAVR procedure. The overall incidence of post-TAVR AF is estimated to be approximately 8%–10%, as supported by an analysis of the Society of Thoracic Surgeons/American College of Cardiology Transcatheter Valve Therapy (STS/ACC TVT) registry by Vora *et al*. [35] and a systematic review and meta-analysis by Ryan *et al*. [36] published in JACC: Cardiovascular Interventions.

AF is defined as a supraventricular tachyarrhythmia characterized by chaotic electrical activity in the atria, leading to ineffective atrial contraction and an irregular ventricular response. The ectopic electrical activity often originates near the pulmonary veins. On ECG, AF is typically identified by the absence of distinct P waves and an irregular R-R interval. The development of AF post-TAVR is clinically significant, as it increases the risk of ischemic stroke, heart failure, and all-cause mortality [37].

A Premature ventricular contraction (PVC) is defined by a wide QRS complex (≥120 ms), abnormal morphology compared to a normal QRS complex, absence of a preceding P wave, and is typically followed by a compensatory pause. Frequent PVCs, defined as more than 10,000–20,000 per day, can lead to a reversible form of cardiomyopathy if the ectopic activity is suppressed. Sustained VT is defined as a ventricular-origin arrhythmia consisting of three or more consecutive complexes at a rate >100 beats per minute lasting ≥30 seconds or requiring termination within 30 seconds due to hemodynamic compromise [38]. Table 1 (Ref. [10, 20, 21, 23, 24, 25, 27, 28, 29, 32, 33, 35, 36, 38, 39, 40]) provides a comprehensive overview of the overall incidence of various conduction abnormalities seen in TAVR.

**Table 1.** **The overall incidence of conduction disturbances as a result of TAVR**.

Conduction disturbance	Overall incidence [Reference]
Left Bundle Branch Block (LBBB)	19%–55% [20]
	Self-expanding CoreValve: 26%–30% [21]
	Self-expanding CoreValve: 18%–65% [23]
	Edwards SAPIEN XT/SAPIEN (3): 4%–30% [23]
	Edwards SAPIEN XT/SAPIEN (3): 3%–30% [24, 25]
High-Degree Atrioventricular Block (HAVB)	10% [10, 20]
	21% [27, 28, 29]
Right Bundle Branch Block (RBBB)	4%–5% [10, 32, 33]
Atrial Fibrillation (AF)	8%–10% [35, 36]
Ventricular Tachycardia (VT)	2% [39]
	9.6% [38]
Premature Ventricular Contractions (PVC)	16% [38]
	48.6% [40]

TAVR, transcatheter aortic valve replacement.

Ventricular arrhythmias have also been observed following TAVR. PVCs were reported in approximately 16% of patients within one year post-procedure, according to a review by Nuche *et al*. [38] published by the HRS. Sustained ventricular tachycardia (VT), though less common, was observed in about 2% of cases [38]. Tempio *et al*. [39] performed 24‑hour Holter monitoring in 146 high‑risk severe AS patients undergoing TAVR and found complex ventricular arrhythmias (non-sustained ventricular tachycardia (NSVT), multifocal PVCs, couplets) in ~49% pre-TAVR. Remarkably, arrhythmia burden fell significantly by one month and continued to decrease by one year post-TAVR (VT prevalence from 9.6% to ~2%) [39]. Moreover, Martinek *et al*. [41] recently reviewed the pathophysiological mechanisms driving ventricular arrhythmias in severe AS prior to intervention, namely LV hypertrophy, fibrosis, ischemia, and conduction disturbances, and emphasized that these arrhythmias should be viewed as markers of advanced disease requiring prompt valve replacement.

A rare but clinically significant arrhythmia post-TAVR is bundle branch reentry (BBR) VT, as described by Belhassen *et al*. [42] in a 74-year-old patient who developed alternating bundle branch block patterns and intermittent wide-complex tachycardia at 187–200 bpm starting three days after valve implantation. Electrophysiological study (EPS) identified a macro-reentrant circuit involving the His–Purkinje system, likely facilitated by conduction system injury during TAVR. Recognition of this phenomenon is crucial, given its rapid rate, hemodynamic impact, and requirement for targeted catheter ablation [42].

The wide range of reported incidence rates for post-TAVR conduction disturbances reflects substantial heterogeneity across published studies. Differences in patient selection, baseline conduction disease, valve platforms and generations, implantation techniques, definitions of conduction abnormalities, and duration and method of post-procedural monitoring limit direct comparison across cohorts. In addition, follow-up intervals vary widely, ranging from in-hospital telemetry to extended ambulatory monitoring, which may underestimate or overestimate late-onset events. As such, the authors recommend reviewers interpret reported incidence as estimates rather than precise event rates.

## 4. Incidence and Clinical Impacts 

Valve types used in TAVR are broadly categorized into balloon-expandable and self-expanding valves. Balloon-expandable valves include early-generation SAPIEN and newer-generation SAPIEN 3 valves, which are generally associated with lower rates of conduction abnormalities. PPM implantation rates for the SAPIEN 3 valve range from 4.0% to 24.0%, while early-generation SAPIEN valves have reported rates as high as 28% [43].

Self-expanding valves include the early-generation CoreValve, newer-generation Evolut R/Pro, and the more recent ACURATE Neo valves. These valves are associated with a higher risk of significant and persistent conduction disturbances. The CoreValve has the highest reported PPM requirement, ranging from 16.3% to 37.7%, while the Evolut R/Pro valves show rates between 14.7% and 26.7% [12, 43]. Self-expanding valves have also been linked to prolonged PR intervals and widened QRS complexes. Notably, the ACURATE Neo valve has demonstrated lower rates of conduction disturbances and PPM requirements compared to other self-expanding valves.

When determining patient candidacy for TAVR, the potential negative clinical consequences and procedural risks must be carefully considered in all cases. Among the most common and significant adverse outcomes are the need for PPM implantation and heart failure-related hospitalizations. PPM placement post-TAVR has been reported in up to 23% of cases within 30 days, depending on valve type [44]. As an invasive procedure, PPM implantation carries additional risks and often necessitates rehospitalization for close cardiac monitoring.

Arrhythmias requiring PPM placement contribute significantly to heart failure-related hospitalizations. These hospitalizations have been reported in 16%–22% of patients within 1–4 years post-TAVR and are primarily attributed to conduction disturbances, paravalvular leak, and procedure-related myocardial injury [45]. A meta-analysis further demonstrated a 32% increased risk of heart failure-related hospitalization in TAVR patients who required PPM implantation [46].

## 5. Risk Factors and Predictors of Permanent Pacemaker Implantation

The ACC highlights several key risk factors and predictors for conduction abnormalities following TAVR. Pre-procedural evaluation is essential for identifying and risk-stratifying patients. ECG serves as a critical first step, and reviewing recent ambulatory cardiac monitoring can help detect bradyarrhythmias or transient AV blocks [10]. Among the most notable predictors, pre- RBBB is strongly associated with the need for PPM implantation post-TAVR. In a multicenter registry, patients with pre-existing RBBB had a 40.1% rate of PPM implantation at 30 days, compared to 13.5% in those without RBBB [47]. These patients also demonstrated higher all-cause and cardiovascular mortality rates (10.2% vs. 6.9%, respectively) [10, 47]. While RBBB is correlated with peri-procedural heart block (up to 24%), its association with delayed heart block (beyond 7 days post-TAVR) has been observed primarily with self-expanding valves [41]. At 18- and 24-month follow-ups, pre-existing RBBB remained the strongest predictor of PPM requirement and was independently associated with increased risk of all-cause and cardiovascular mortality [47, 48].

The role of pre-existing LBBB has also been explored. However, current evidence does not support a correlation between LBBB and increased risk of immediate or delayed cardiovascular or all-cause mortality. Research on new-onset LBBB post-TAVR presents conflicting findings regarding the necessity of PPM implantation. No statistically significant differences in cardiovascular mortality, hospitalizations, or heart failure development have been observed at 1-, 6-, and 12-month intervals [49, 50]. Further data is needed to assess long-term safety in patients with new-onset LBBB, with or without pacemaker placement.

Evidence regarding standardized PPM placement at the time of TAVR for patients with pre-existing conduction abnormalities such as variable AV block, prolonged PR interval, or prolonged HV interval (>55 ms or >70 ms) is less robust. Emerging studies suggest that intraoperative monitoring and post-TAVR measurements of these conduction parameters may correlate with mortality risk and pacemaker necessity.

Valve type also plays a role in conduction disturbance risk. Pre-dilation has shown a modest association with heart block, but self-expanding valves consistently demonstrate higher PPM implantation rates compared to balloon-expandable valves. For example, the CoreValve prosthesis has a PPM rate of 25.8% versus 6.5% for the Edwards SAPIEN valve, with an odds ratio of 4.91 (95% confidence interval (CI): 4.12–5.86; *p* < 0.001) [10, 51, 52, 53]. Multiple studies, including prospective and retrospective trials, meta-analyses, and large registries, report RRs ranging from 1.8 to 3.0, attributed to the greater radial force and deeper implantation depth of self-expanding valves [53, 54, 55].

An elevated prosthesis-to-LVOT diameter ratio has been associated with increased PPM implantation risk. However, this correlation should be interpreted cautiously in studies involving balloon-expandable valves due to the lack of reported implantation depth [10, 56]. When implantation depth exceeds the length of the MS, the odds of conduction disturbance rise significantly. The MS is located at the aortic root between the right coronary and non-coronary cusps and houses the penetrating bundle of His, making it a high-risk site for injury during TAVR due to its small size and critical anatomical location. Patients with an MS length <2 mm are at significantly increased risk for PPM implantation [57]. In a retrospective study by Hamdan *et al*. [58], MS length was identified as a strong predictor of PPM requirement prior to TAVR. Post-TAVR, the most powerful predictor was the ratio of MS length to implantation depth [58].

Other anatomical variables, including calcifications and annulus perimeter/eccentricity, also correlate with increased risk of conduction disturbances. Specifically, calcifications at the right coronary and non-coronary cusps, LVOT, basal septum, and aortic root are independent imaging biomarkers associated with higher risk of conductive injury [48, 54, 57]. While multivariable risk models can help estimate pacemaker risk after TAVR, they are often difficult to apply in everyday clinical practice. In reality, clinicians typically rely on a stepwise assessment using readily available information, including baseline ECG findings (such as pre-existing RBBB or PR prolongation), pre-procedural cardiac CT measurements of membranous septal length, LVOT size, and calcium distribution, as well as careful attention to implantation depth during the procedure. Rather than using formal algorithms, these factors are often considered with each other to guide shared decision-making, procedural planning, and the intensity of post-TAVR rhythm monitoring. In patients felt to be at higher risk, this approach may favor closer surveillance or early electrophysiology involvement rather than routine prophylactic pacemaker implantation. Table 2 shows a comprehensive overview of PPM rates after TAVR that vary significantly by valve type, with self-expanding and mechanically expandable valves generally associated with higher rates than balloon-expandable valves. New-generation devices have reduced, but not eliminated, this risk. Interpretation of valve-specific pacemaker implantation rates should account for temporal trends and operator learning curves. Earlier-generation devices were frequently implanted during the initial adoption phase of TAVR, when implantation techniques, depth control, and imaging integration were less standardized. Subsequent refinements in device design, delivery systems, implantation strategies, and operator experience have been associated with progressive reductions in pacemaker rates, even within the same valve platform. These factors should be considered when extrapolating historical data to contemporary clinical practice.

**Table 2.** **Overview of PPM incidence based on different valve types**.

Valve type/Device	PPM incidence range (%)	Models	Notes
Balloon-expandable (BE)	4%–14%	SAPIEN XT, SAPIEN 3, PARTNER 2 S3	Lower risk; SAPIEN 3: 12.5% (PARTNER 2 S3); anatomical and procedural factors matter
Self-expanding (SE)	11%–25%	CoreValve, Evolut R/PRO, Acurate Neo	Higher risk; CoreValve: up to 30%; Evolut R/PRO: 14%–17%; Acurate Neo: 8%–9%
Mechanically expandable (ME)	25%–35%	Lotus, Direct Flow	Highest risk; Lotus: up to 34%; ME valves OR 3.48 vs BE valves for PPM implantation
Valve-in-valve (ViV) TAVR	3.7%–7.4%	Evolut R/Pro, CoreValve, VIVID	New-generation THVs: 4.7%; Evolut R/Pro: 3.7%; CoreValve: 9.0%
All TAVR (overall pooled)	10%–22%	All models	Meta-analyses, wide range due to device, patient, and center variability

PPM, permanent pacemaker; THV, transcatheter heart valves; VIVID, Valve-in-Valve International Data Registry.

## 6. Risk Stratifications and Monitoring 

Continuous rhythm monitoring plays an important role in evaluating both peri-procedural and delayed heart blocks following TAVR. As previously discussed, pre-existing conduction abnormalities are important considerations in all TAVR patients. The strongest predictors for eventual PPM implantation include:

-Prolonged PR interval following valve deployment

-HV interval duration >80–90 ms post-TAVR

-Prolonged AV nodal conduction

-Lower atrial pacing rates required to induce second-degree Mobitz I (Wenckebach) AV block [10]

Multivariable analyses and retrospective studies have identified prolonged PR interval as a significant predictor of pacing requirement, including the development of delayed advanced conduction abnormalities occurring more than 48 hours post-procedure [58, 59, 60]. One of the most effective tools for predicting the need for PPM implantation is rapid atrial pacing. Among all risk factors, this method has the highest negative predictive value (NPV). In a study by Krishnaswamy *et al*. [61], 284 patients underwent rapid atrial pacing (70–120 bpm) via a temporary pacemaker lead placed in the right atrium following valve deployment. Patients who developed Wenckebach AV block during pacing had a significantly higher rate of PPM implantation (13.1%) compared to those who did not (1.3%, *p* < 0.001) [61]. The NPV for patients who did not develop Wenckebach was 98.7%. This predictive value remained consistent across valve types, including both self-expanding and balloon-expandable valves. The study also included patients with pre-existing and newly diagnosed RBBB and LBBB.

The 2020 ACC Expert Consensus Panel recommends a risk-stratified approach to post-operative monitoring for conduction disturbances, based on pre-TAVR risk factors and peri-procedural findings. Three clinical scenarios are outlined:

-No new or worsened conduction disturbances (e.g., <20 ms change in PR/QRS intervals)

-New bundle branch block or ≥10% increase in PR/QRS interval duration

-Peri-operative transient or persistent complete heart block [10]

For patients with new or worsened conduction disturbances, the panel recommends a minimum of 48 hours of inpatient monitoring, extending up to 7 days if ambulatory monitoring is not planned. If ambulatory monitoring is used, a minimum duration of 14 days post-discharge is advised, along with specific criteria for response and patient safety.

For patients without new or worsened conduction disturbances, 48 hours of inpatient monitoring is recommended, with discharge considered if the ECG remains stable, as the risk of delayed heart block is <1%. In cases of perioperative transient or persistent AV block, especially in patients with pre-existing RBBB, the panel recommends placement of a temporary pacemaker lead for at least 24 hours, with strong consideration for PPM implantation prior to discharge [10].

## 7. Indications for PPM Following TAVR 

PPM implantation after TAVR is primarily indicated in cases of persistent HAVB, recurrent transient HAVB, or new conduction abnormalities in patients with pre- RBBB. Current guidelines recommend PPM placement for patients who develop sustained or recurrent HAVB during or after TAVR, regardless of symptom presentation. In patients with baseline RBBB, the threshold for pacing is particularly low, as transient or persistent AV block in this population carries a substantial risk of long-term pacing dependence. Emerging evidence also suggests that new or persistent LBBB may be a marker for increased late PPM requirement, although some cases may resolve within 6–12 months [10, 20].

Most PPMs are implanted within the first week following TAVR, with a median time of approximately 3 days. Over 60% of cases occur intra-procedurally or within the first 24 hours, while the remainder typically occur within 7 days of valve implantation [62, 63]. A minority of patients, particularly those with new LBBB or pre-existing RBBB, may present with delayed HAVB requiring pacing weeks after hospital discharge [20]. To address transient conduction disturbances, the ACC recommends temporary pacing and close rhythm surveillance for at least 24 hours before committing to PPM implantation. Ideally, TAVR and PPM procedures should be separated to allow for informed consent and procedural optimization [10, 20].

There is substantial variability in PPM implantation practices across institutions. Reported rates for new-generation valves range from as low as 2% to over 30% [35]. This variability reflects differences in institutional thresholds, monitoring protocols, operator experience, valve selection, and procedural techniques such as implantation depth and valve oversizing. For example, balloon-expandable valves (e.g., SAPIEN 3) are associated with lower PPM rates (6%–13%), whereas self-expanding valves (e.g., CoreValve, Evolut PRO) have higher rates (15%–25%) [62, 63]. Although high-volume centers with standardized protocols may achieve more consistent outcomes, no single strategy has proven universally superior. Professional societies emphasize tailoring protocols to local expertise and available resources [10, 64].

Long-term ventricular pacing dependence is common among patients who receive a PPM post-TAVR, with rates ranging from 50% to 80% in follow-up studies [62, 63, 64]. The likelihood of persistent dependence is highest in patients with pre-existing conduction disease or those who develop sustained AV block after valve implantation [10]. While PPM implantation does not consistently correlate with increased long-term mortality, it has been associated with subtle adverse cardiac remodeling. This includes a modest reduction in left ventricular ejection fraction. In some cohorts, higher one-year mortality has been observed, particularly among patients with impaired baseline systolic function [62, 63]. These findings underscore the importance of careful patient selection and vigilant follow-up.

Management of conduction disturbances post-TAVR is guided by society recommendations and supported by an expanding body of clinical evidence. The ACC emphasizes PPM implantation for patients with persistent or recurrent HAVB, regardless of symptoms. Patients with pre-existing RBBB who develop new or worsening conduction disturbances are considered high-risk and warrant early pacing consideration. The ACC also stresses the importance of shared decision-making, patient counseling, and individualized assessment, especially in those with impaired left ventricular ejection fraction, given the unpredictable course of conduction disease following TAVR [10]. Electrophysiologic study-guided strategies, including assessment of HV interval prolongation or inducible infra-Hisian block, may further refine risk stratification and help identify patients who can safely defer PPM implantation. Importantly, shared decision-making is central in borderline cases, balancing the risks of delayed high-grade block against the potential for recovery of conduction and avoidance of lifelong pacing.

The HRS, in collaboration with the ACC and AHA, similarly recommends PPM implantation for symptomatic bradycardia or HAVB after TAVR. These guidelines also highlight the role of physiologic pacing techniques, such as His-bundle or LBB area pacing, to reduce the long-term risk of pacing-induced cardiomyopathy in patients expected to require frequent ventricular pacing by bringing back synchronized ventricular contraction [20]. The European Society of Cardiology (ESC) aligns closely with these principles, supporting PPM implantation in cases of persistent high-grade AV block or high-risk conduction abnormalities, while endorsing close monitoring and individualized care for transient or less severe conduction disturbances [65, 66]. Table 3 presents a summary of societal recommendations regarding PPM indications.

**Table 3.** **Summary of PPM indication based on various societal guidelines**.

Society	Indication for PPM	Timing	Special considerations	Rationale
ACC	Persistent or recurrent high-grade AV block; new/worsening conduction in pre-existing RBBB	Usually within 7 days post-TAVR	Shared decision-making, pre-procedure counseling, and individualized risk	Prevent sudden death, manage unpredictable conduction disease
HRS (with ACC/AHA)	Symptomatic bradycardia or high-grade AV block; frequent pacing needs	Early post-TAVR if persistent block	Prefer physiological pacing if >40% ventricular pacing expected	Reduce pacing-induced heart failure, patient-centered care
ESC	Persistent high-grade AV block; high-risk conduction disturbances	Early post-TAVR if persistent block	Close monitoring for transient/less severe cases	Prevent progression to complete block, sudden death

Recent studies reinforce the importance of risk stratification. Patients with baseline conduction disease, including RBBB or bifascicular block, are at increased risk of post-TAVR PPM, particularly when self-expanding valves or deep implantations are used [67]. For patients with borderline indications (e.g., transient high-grade AV block or new-onset LBBB), extended inpatient telemetry for 24–72 hours and ambulatory ECG monitoring are recommended to detect delayed progression [62]. EPS-guided strategies can further refine decision-making, demonstrating good accuracy in identifying patients who may safely avoid PPM [68].

**Fig. 2.**
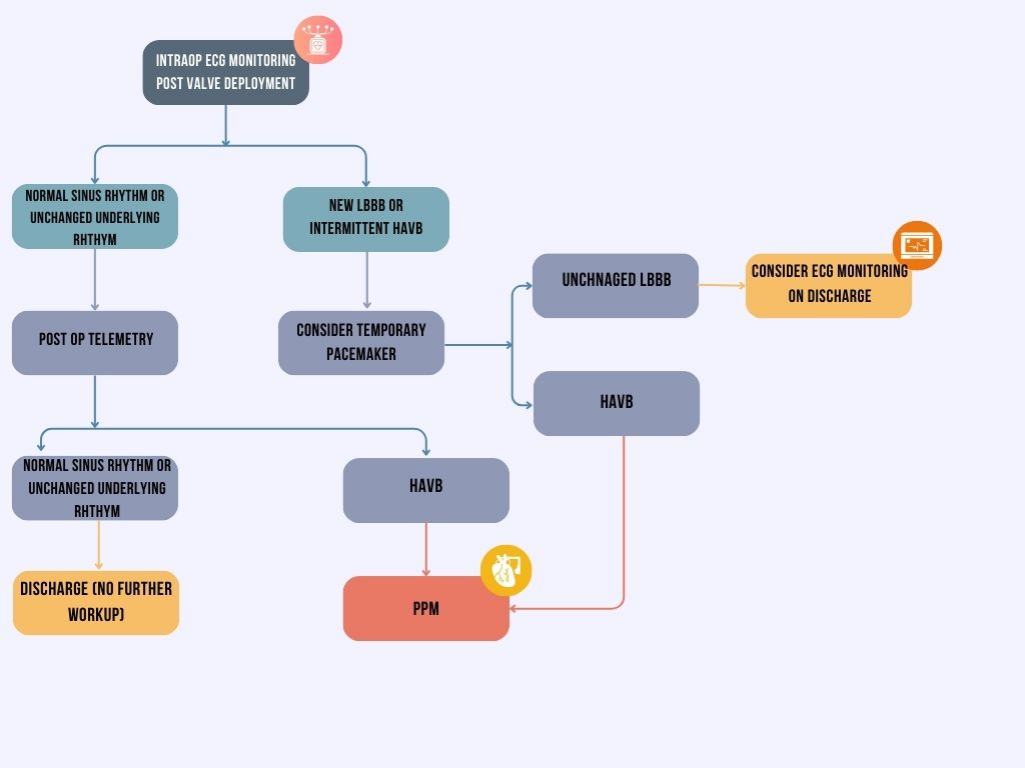
**Algorithmic approach to post-TAVR conduction disturbances**. Figure created with Canva.

Valve selection and implantation techniques are integral to minimizing conduction disturbances. Balloon-expandable valves and attention to shallow implantation depth are consistently associated with lower PPM rates compared with self-expanding valves and deeper deployment [67, 69]. In high-risk patients, consideration of valve type and procedural modification may mitigate subsequent pacing requirements. Given the potential for late-onset conduction disease, particularly in patients with new or persistent LBBB, long-term rhythm follow-up is essential. Ambulatory monitoring, coupled with patient education regarding symptoms of bradycardia, forms the basis of extended surveillance [62, 70]. Across guidelines and consensus documents, a recurring theme is the importance of a heart team multidisciplinary approach that incorporates individualized risk assessment, procedural planning, and structured post-procedural monitoring. This multidisciplinary strategy is considered best practice to optimize patient outcomes [19, 62]. Fig. 2 presents a stepwise algorithm for evaluating and managing conduction disturbances following TAVR.

## 8. Future Directions 

Current practice for PPM implantation after TAVR varies considerably among institutions, particularly regarding the management of new bundle branch block and transient high-grade AV block. The ACC consensus pathway aims to address this heterogeneity and emphasizes the importance of developing evidence-based, standardized algorithms in order to minimize unnecessary PPM implantation while ensuring patient safety in TAVR patients [10, 30]. Cardiac CT and ECG-gated CT angiography have become essential for pre-procedural risk stratification, enabling detailed evaluation of factors such as membranous septal length, annular and LVOT calcification, and valve-anatomy relationships, which are all strong predictors of post-TAVR conduction disturbances [52, 54, 71, 72]. Patient-specific computational modeling is also advancing the field. The GUIDE-TAVI trial is assessing whether pre-procedural simulations of device–anatomy interactions can improve prediction of conduction risk and optimize valve implantation strategy [73]. Adapting management to an individual’s anatomy and risk profile is an essential step towards addressing this procedural complication concern. By integrating clinical characteristics, ECG findings, and CT-derived anatomical markers, clinicians can adjust valve selection, implantation depth, and pacing strategy to reduce PPM rates [27, 54, 67, 70]. Such personalized approaches will be particularly important as TAVR expands to younger and lower-risk populations with newer studies [54, 67]. These trials are expected to support a more standardized and evidence-based post-TAVR monitoring protocol by refining patient selection for early versus deferred pacemaker implantation as well.

AI-based tools are being explored to enhance pre- and intra-procedural decision-making. Early models applying machine learning to imaging and ECG data show potential for improving the prediction of conduction disturbances and enabling real-time procedural adjustments [72]. While still in development, AI-driven risk stratification could provide reproducible and individualized guidance for pacing decisions. Several prospective trials aim to inform future practice. The GUIDE-TAVI trial is evaluating the clinical utility of patient-specific computer simulation, while the CONDUCT-TAVI study is prospectively testing invasive electrophysiology, CT, and ECG predictors of high-grade AV block with continuous monitoring for delayed events [27, 73]. Although not focused specifically on conduction disturbances, recent multicenter work by Wang *et al*. [74] demonstrates how explainable machine learning models integrating imaging, electrocardiographic, and clinical data can enable early post-TAVR risk stratification and prognosis prediction. Such frameworks may be adaptable to future conduction-specific prediction models and support individualized rhythm surveillance and management strategies after TAVR [74].

These efforts are expected to shape standardized, personalized pathways for managing conduction disturbances post-TAVR. From a regulatory perspective, many AI-driven decision-support tools are likely to require formal evaluation as medical devices. These will require oversight from regulatory agencies such as the U.S. Food and Drug Administration. Therefore, demonstration of clinical benefit, reproducibility, and safety will be essential before widespread adoption. As such, near-term implementation is most likely to occur at high-volume centers with established imaging infrastructure and multidisciplinary expertise.

As TAVR expands into younger and lower-risk populations, an increasing proportion of these patients have BAV anatomy. BAV is characterized by asymmetric annular and LVOT calcification, elliptical annular geometry, and frequent raphe involvement. These factors can alter valve expansion and increase mechanical stress on the conduction system. These anatomical differences raise the question of whether conduction disturbances after TAVR occur more frequently or behave differently in BAV compared with tricuspid aortic valve (TAV) disease. Available data suggest that overall rates of new conduction abnormalities and PPM implantation in BAV patients are often similar to those seen in TAV patients when contemporary valve platforms and optimized implantation techniques are used [75, 76]. Future prospective studies using standardized imaging metrics and uniform definitions of conduction outcomes are needed to clarify whether BAV anatomy independently modifies conduction risk and long-term pacing dependence. A better understanding of these differences may help refine patient selection, procedural planning, and post-TAVR rhythm surveillance in this growing population.

The cost implications of managing conduction disturbances after TAVR also deserve consideration. Approaches such as extended inpatient monitoring, ambulatory rhythm surveillance, electrophysiology testing, and PPM implantation differ substantially in both cost and resource use. Although closer monitoring may improve safety in higher-risk patients, it may offer limited added value in lower-risk individuals while increasing overall healthcare costs. Future studies that specifically examine the cost-effectiveness of different monitoring and pacing strategies will be important to guide practice guidelines and support more efficient, value-based care in structural heart disease.

## 9. Limitations

Several limitations should be acknowledged. As a narrative review, this work does not employ a formal systematic review methodology or meta-analytic techniques, which limit quantitative comparison across studies. Additionally, the rapid evolution of TAVR technologies, delivery systems, and implantation techniques introduces significant temporal heterogeneity across the literature. Earlier studies frequently reflect first- or second-generation devices and implantation strategies that are no longer representative of contemporary practice. As a result, reported incidence rates of conduction disturbances and pacemaker implantation from older cohorts may overestimate risk relative to current-generation valves and refined procedural techniques

## 10. Conclusion

TAVR has revolutionized the management of severe aortic stenosis by offering a less invasive option with comparable or superior outcomes to SAVR in many patient populations. However, conduction disturbances remain a significant concern with implications for morbidity, mortality, and long-term cardiac function. These complications arise from the close anatomical relationship between the aortic valve and the conduction system and are exacerbated by procedural factors such as valve type and implantation depth. While advancements in valve design have reduced some risks, persistent conduction issues highlight the need for improved pre-procedural assessment, individualized valve selection, and post-procedural monitoring. Future research should focus on refining risk prediction models, developing conduction-sparing valve technologies, and establishing consensus guidelines for monitoring and managing conduction disturbances.

## Product and Device Identification

The THV referenced in this review include balloon-expandable valves such as the SAPIEN XT and SAPIEN 3 (Edwards Lifesciences; Irvine, CA, USA) and self-expanding valves, including the CoreValve and Evolut R/Pro systems (Medtronic; Minneapolis, MN, USA), as well as the ACURATE Neo valve (Boston Scientific; Marlborough, MA, USA). Device and product specifications are reported as described in the original cited studies. No manufacturer-specific comparisons beyond published data were performed by the authors.

## References

[1] Otto CM, Kumbhani DJ, Alexander KP, Calhoon JH, Desai MY, Kaul S (2017). 2017 ACC Expert Consensus Decision Pathway for Transcatheter Aortic Valve Replacement in the Management of Adults With Aortic Stenosis: A Report of the American College of Cardiology Task Force on Clinical Expert Consensus Documents. *Journal of the American College of Cardiology*.

[2] Baumgartner H, Hung J, Bermejo J, Chambers JB, Edvardsen T, Goldstein S (2017). Recommendations on the Echocardiographic Assessment of Aortic Valve Stenosis: A Focused Update from the European Association of Cardiovascular Imaging and the American Society of Echocardiography. *Journal of the American Society of Echocardiography: Official Publication of the American Society of Echocardiography*.

[3] Otto CM, Newby DE, Hillis GS (2024). Calcific Aortic Stenosis: A Review. *JAMA*.

[4] Davidson LJ, Davidson CJ (2021). Transcatheter Treatment of Valvular Heart Disease: A Review. *JAMA*.

[5] Fang F, Tang J, Zhao Y, He J, Xu P, Faramand A (2019). Transcatheter aortic valve implantation versus surgical aortic valve replacement in patients at low and intermediate risk: A risk specific meta-analysis of randomized controlled trials. *PloS One*.

[6] Forrest JK, Deeb GM, Yakubov SJ, Rovin JD, Mumtaz M, Gada H (2022). 2-Year Outcomes After Transcatheter Versus Surgical Aortic Valve Replacement in Low-Risk Patients. *Journal of the American College of Cardiology*.

[7] Caminiti R, Ielasi A, Vetta G, Parlavecchio A, Rocca DGD, Glauber M (2024). Long-Term Results Following Transcatheter Versus Surgical Aortic Valve Replacement in Low-Risk Patients With Severe Aortic Stenosis: A Systematic Review and Meta-Analysis of Randomized Trials. *The American Journal of Cardiology*.

[8] Writing Committee Members, Otto CM, Nishimura RA, Bonow RO, Carabello BA, Erwin JP (2021). 2020 ACC/AHA Guideline for the Management of Patients With Valvular Heart Disease: A Report of the American College of Cardiology/American Heart Association Joint Committee on Clinical Practice Guidelines. *Journal of the American College of Cardiology*.

[9] Boskovski MT, Nguyen TC, McCabe JM, Kaneko T (2020). Outcomes of Transcatheter Aortic Valve Replacement in Patients With Severe Aortic Stenosis: A Review of a Disruptive Technology in Aortic Valve Surgery. *JAMA Surgery*.

[10] Lilly SM, Deshmukh AJ, Epstein AE, Ricciardi MJ, Shreenivas S, Velagapudi P (2020). 2020 ACC Expert Consensus Decision Pathway on Management of Conduction Disturbances in Patients Undergoing Transcatheter Aortic Valve Replacement: A Report of the American College of Cardiology Solution Set Oversight Committee. *Journal of the American College of Cardiology*.

[11] De Almeida MC, Sanchez-Quintana D, Anderson RH (2021). The membranous septum revisited: A glimpse of our anatomical past. *Clinical Anatomy (New York, N.Y.)*.

[12] Castro-Mejía AF, Amat-Santos I, Ortega-Armas ME, Baz JA, Moreno R, Diaz JF (2022). Development of atrioventricular and intraventricular conduction disturbances in patients undergoing transcatheter aortic valve replacement with new generation self-expanding valves: A real world multicenter analysis. *International Journal of Cardiology*.

[13] Mori S, Tretter JT, Toba T, Izawa Y, Tahara N, Nishii T (2018). Relationship between the membranous septum and the virtual basal ring of the aortic root in candidates for transcatheter implantation of the aortic valve. *Clinical Anatomy (New York, N.Y.)*.

[14] Jilaihawi H, Chen M, Webb J, Himbert D, Ruiz CE, Rodés-Cabau J (2016). A Bicuspid Aortic Valve Imaging Classification for the TAVR Era. *JACC: Cardiovascular Imaging*.

[15] Maeno Y, Abramowitz Y, Kawamori H, Kazuno Y, Kubo S, Takahashi N (2017). A Highly Predictive Risk Model for Pacemaker Implantation After TAVR. *JACC. Cardiovascular Imaging*.

[16] Tretter JT, Eleid MF, Bedogni F, Rodés-Cabau J, Regueiro A, Testa L (2025). Preprocedural CT and ECG Markers for Predicting Post-TAVR Pacemaker Requirement in High-Risk Patients. *Structural Heart: the Journal of the Heart Team*.

[17] Kawashima T, Sasaki H (2005). A macroscopic anatomical investigation of atrioventricular bundle locational variation relative to the membranous part of the ventricular septum in elderly human hearts. *Surgical and Radiologic Anatomy: SRA*.

[18] Nakashima M, Jilaihawi H (2021). Conduction Disturbances and Pacing in Transcatheter Aortic Valve Replacement. *Interventional Cardiology Clinics*.

[19] Papa A, Serban T, Strebel I, Knecht S, Isenegger C, Nestelberger T (2023). Impact of implantation depth and calcium burden on infranodal conduction delay after transcatheter aortic valve replacement. *Heart Rhythm O2*.

[20] Kusumoto FM, Schoenfeld MH, Barrett C, Edgerton JR, Ellenbogen KA, Gold MR (2019). 2018 ACC/AHA/HRS Guideline on the Evaluation and Management of Patients With Bradycardia and Cardiac Conduction Delay: A Report of the American College of Cardiology/American Heart Association Task Force on Clinical Practice Guidelines and the Heart Rhythm Society. *Circulation*.

[21] Alzu’bi H, Rmilah AA, Bahmad HF, Urina-Jassir D, Elajami MK, Rogers E (2025). Predictors of Post-TAVR Left Bundle Branch Block: A Systematic Review and Meta-Analysis. *Journal of Cardiovascular Electrophysiology*.

[22] Isogai T, Dykun I, Agrawal A, Shekhar S, Tarakji KG, Wazni OM (2022). Early Resolution of New-Onset Left Bundle Branch Block After Transcatheter Aortic Valve Implantation With the SAPIEN 3 Valve. *The American Journal of Cardiology*.

[23] Auffret V, Puri R, Urena M, Chamandi C, Rodriguez-Gabella T, Philippon F (2017). Conduction Disturbances After Transcatheter Aortic Valve Replacement: Current Status and Future Perspectives. *Circulation*.

[24] Nazif TM, Chen S, George I, Dizon JM, Hahn RT, Crowley A (2019). New-onset left bundle branch block after transcatheter aortic valve replacement is associated with adverse long-term clinical outcomes in intermediate-risk patients: an analysis from the PARTNER II trial. *European Heart Journal*.

[25] Nazif TM, Williams MR, Hahn RT, Kapadia S, Babaliaros V, Rodés-Cabau J (2014). Clinical implications of new-onset left bundle branch block after transcatheter aortic valve replacement: analysis of the PARTNER experience. *European Heart Journal*.

[26] Massoullié G, Ploux S, Souteyrand G, Mondoly P, Pereira B, Amabile N (2023). Incidence and management of atrioventricular conduction disorders in new-onset left bundle branch block after TAVI: A prospective multicenter study. *Heart Rhythm*.

[27] Rao K, Bhatia K, Chan B, Cowan M, Saad N, Baer A (2023). Prospective observational study on the accuracy of predictors of high-grade atrioventricular conduction block after transcatheter aortic valve implantation (CONDUCT-TAVI): study protocol, background and significance. *BMJ Open*.

[28] El-Sabawi B, Welle GA, Cha YM, Espinosa RE, Gulati R, Sandhu GS (2021). Temporal Incidence and Predictors of High-Grade Atrioventricular Block After Transcatheter Aortic Valve Replacement. *Journal of the American Heart Association*.

[29] Reiter C, Lambert T, Kellermair J, Blessberger H, Fellner A, Nahler A (2021). Delayed Total Atrioventricular Block After Transcatheter Aortic Valve Replacement Assessed by Implantable Loop Recorders. *JACC. Cardiovascular Interventions*.

[30] Ream K, Sandhu A, Valle J, Weber R, Kaizer A, Wiktor DM (2019). Ambulatory Rhythm Monitoring to Detect Late High-Grade Atrioventricular Block Following Transcatheter Aortic Valve Replacement. *Journal of the American College of Cardiology*.

[31] Faroux L, Chen S, Muntané-Carol G, Regueiro A, Philippon F, Sondergaard L (2020). Clinical impact of conduction disturbances in transcatheter aortic valve replacement recipients: a systematic review and meta-analysis. *European Heart Journal*.

[32] Kikuchi S, Minamimoto Y, Matsushita K, Cho T, Terasaka K, Hanajima Y (2024). Impact of New-Onset Right Bundle-Branch Block After Transcatheter Aortic Valve Replacement on Permanent Pacemaker Implantation. *Journal of the American Heart Association*.

[33] Tan NY, Adedinsewo D, El Sabbagh A, Sayed Ahmed AF, Carolina Morales-Lara A, Wieczorek M (2024). Incidence and Outcomes of New-Onset Right Bundle Branch Block Following Transcatheter Aortic Valve Replacement. *Circulation. Arrhythmia and Electrophysiology*.

[34] Michowitz Y, Yagel O, Shrem M, Elbaz-Greener G, Tovia-Brodie O, Goldenberg GR (2025). New-Onset RBBB After Transcatheter Aortic Valve Replacement: Incidence and Risk Factors for Permanent Pacemaker Implantation. *JACC. Clinical Electrophysiology*.

[35] Vora AN, Dai D, Matsuoka R, Harrison JK, Hughes GC, Sherwood MW (2018). Incidence, Management, and Associated Clinical Outcomes of New-Onset Atrial Fibrillation Following Transcatheter Aortic Valve Replacement: An Analysis From the STS/ACC TVT Registry. *JACC. Cardiovascular Interventions*.

[36] Ryan T, Grindal A, Jinah R, Um KJ, Vadakken ME, Pandey A (2022). New-Onset Atrial Fibrillation After Transcatheter Aortic Valve Replacement: A Systematic Review and Meta-Analysis. *JACC. Cardiovascular Interventions*.

[37] Joglar JA, Chung MK, Armbruster AL, Benjamin EJ, Chyou JY, Cronin EM (2024). 2023 ACC/AHA/ACCP/HRS Guideline for the Diagnosis and Management of Atrial Fibrillation: A Report of the American College of Cardiology/American Heart Association Joint Committee on Clinical Practice Guidelines. *Circulation*.

[38] Nuche J, Panagides V, Nault I, Mesnier J, Paradis JM, de Larochellière R (2022). Incidence and clinical impact of tachyarrhythmic events following transcatheter aortic valve replacement: A review. *Heart Rhythm*.

[39] Tempio D, Pruiti GP, Conti S, Romano SA, Tavano E, Capodanno D (2015). Ventricular arrhythmias in aortic valve stenosis before and after transcatheter aortic valve implantation. *Europace: European Pacing, Arrhythmias, and Cardiac Electrophysiology: Journal of the Working Groups on Cardiac Pacing, Arrhythmias, and Cardiac Cellular Electrophysiology of the European Society of Cardiology*.

[40] Al-Khatib SM, Stevenson WG, Ackerman MJ, Bryant WJ, Callans DJ, Curtis AB (2018). 2017 AHA/ACC/HRS Guideline for Management of Patients With Ventricular Arrhythmias and the Prevention of Sudden Cardiac Death: A Report of the American College of Cardiology/American Heart Association Task Force on Clinical Practice Guidelines and the Heart Rhythm Society. *Journal of the American College of Cardiology*.

[41] Martinek M, Jiravsky O, Cesnakova Konecna A, Adamek J, Chovancik J, Sknouril L (2025). Ventricular Arrhythmias in Severe Aortic Stenosis Prior to Aortic Valve Replacement: A Literature Review. *Medicina (Kaunas, Lithuania)*.

[42] Belhassen B, Shauer A, Biton Y (2021). Left Bundle-Branch Block Tachycardia After Transcatheter Aortic Valve Replacement. *Circulation*.

[43] van Rosendael PJ, Delgado V, Bax JJ (2018). Pacemaker implantation rate after transcatheter aortic valve implantation with early and new-generation devices: a systematic review. *European Heart Journal*.

[44] Badertscher P, Stortecky S, Serban T, Knecht S, Heg D, Tueller D (2025). Long-Term Outcomes of Patients Requiring Pacemaker Implantation After Transcatheter Aortic Valve Replacement: The SwissTAVI Registry. *JACC. Cardiovascular Interventions*.

[45] Mengi S, Januzzi JL, Cavalcante JL, Avvedimento M, Galhardo A, Bernier M (2024). Aortic Stenosis, Heart Failure, and Aortic Valve Replacement. *JAMA Cardiology*.

[46] Zito A, Princi G, Lombardi M, D’Amario D, Vergallo R, Aurigemma C (2022). Long-term clinical impact of permanent pacemaker implantation in patients undergoing transcatheter aortic valve implantation: a systematic review and meta-analysis. *Europace: European Pacing, Arrhythmias, and Cardiac Electrophysiology: Journal of the Working Groups on Cardiac Pacing, Arrhythmias, and Cardiac Cellular Electrophysiology of the European Society of Cardiology*.

[47] Auffret V, Webb JG, Eltchaninoff H, Muñoz-García AJ, Himbert D, Tamburino C (2017). Clinical Impact of Baseline Right Bundle Branch Block in Patients Undergoing Transcatheter Aortic Valve Replacement. *JACC. Cardiovascular Interventions*.

[48] Sugiyama Y, Miyashita H, Yokoyama H, Ochiai T, Shishido K, Jalanko M (2024). Risk Assessment of Permanent Pacemaker Implantation After Transcatheter Aortic Valve Implantation in Patients With Preexisting Right Bundle Branch Block. *The American Journal of Cardiology*.

[49] Mangieri A, Montalto C, Pagnesi M, Lanzillo G, Demir O, Testa L (2018). TAVI and Post Procedural Cardiac Conduction Abnormalities. *Frontiers in Cardiovascular Medicine*.

[50] Urena M, Webb JG, Cheema A, Serra V, Toggweiler S, Barbanti M (2014). Impact of new-onset persistent left bundle branch block on late clinical outcomes in patients undergoing transcatheter aortic valve implantation with a balloon-expandable valve. *JACC. Cardiovascular Interventions*.

[51] Bianchini F, Bianchini E, Romagnoli E, Aurigemma C, Zito A, Busco M (2024). Anatomical Annulus Predictors of New Permanent Pacemaker Implantation Risk After Balloon-Expandable Transcatheter Aortic Valve Implantation. *The American Journal of Cardiology*.

[52] Maier O, Piayda K, Afzal S, Polzin A, Westenfeld R, Jung C (2021). Computed tomography derived predictors of permanent pacemaker implantation after transcatheter aortic valve replacement: A meta-analysis. *Catheterization and Cardiovascular Interventions: Official Journal of the Society for Cardiac Angiography & Interventions*.

[53] Mahajan S, Gupta R, Malik AH, Mahajan P, Aedma SK, Aronow WS (2021). Predictors of permanent pacemaker insertion after TAVR: A systematic review and updated meta-analysis. *Journal of Cardiovascular Electrophysiology*.

[54] Jung S, Kondruweit M, Marwan M, Achenbach S (2025). Anatomical and Functional Predictors of Permanent Pacemaker Implantation After Transcatheter Aortic Valve Implantation. *Journal of the American Heart Association*.

[55] Vlastra W, Chandrasekhar J, Muñoz-Garcia AJ, Tchétché D, de Brito FS, Barbanti M (2019). Comparison of balloon-expandable vs. self-expandable valves in patients undergoing transfemoral transcatheter aortic valve implantation: from the CENTER-collaboration. *European Heart Journal*.

[56] Nazif TM, Dizon JM, Hahn RT, Xu K, Babaliaros V, Douglas PS (2015). Predictors and clinical outcomes of permanent pacemaker implantation after transcatheter aortic valve replacement: the PARTNER (Placement of AoRtic TraNscathetER Valves) trial and registry. *JACC. Cardiovascular Interventions*.

[57] Chen YH, Chang HH, Liao TW, Leu HB, Chen IM, Chen PL (2022). Membranous septum length predicts conduction disturbances following transcatheter aortic valve replacement. *The Journal of Thoracic and Cardiovascular Surgery*.

[58] Hamdan A, Guetta V, Klempfner R, Konen E, Raanani E, Glikson M (2015). Inverse Relationship Between Membranous Septal Length and the Risk of Atrioventricular Block in Patients Undergoing Transcatheter Aortic Valve Implantation. *JACC. Cardiovascular Interventions*.

[59] Tichelbäcker T, Bergau L, Puls M, Friede T, Mütze T, Maier LS (2018). Insights into permanent pacemaker implantation following TAVR in a real-world cohort. *PloS One*.

[60] Mangieri A, Lanzillo G, Bertoldi L, Jabbour RJ, Regazzoli D, Ancona MB (2018). Predictors of Advanced Conduction Disturbances Requiring a Late (≥48 H) Permanent Pacemaker Following Transcatheter Aortic Valve Replacement. *JACC. Cardiovascular Interventions*.

[61] Krishnaswamy A, Sammour Y, Mangieri A, Kadri A, Karrthik A, Banerjee K (2020). The Utility of Rapid Atrial Pacing Immediately Post-TAVR to Predict the Need for Pacemaker Implantation. *JACC. Cardiovascular Interventions*.

[62] Mazzella AJ, Sanders M, Yang H, Li Q, Vavalle JP, Gehi A (2021). Predicting need for pacemaker implantation early and late after transcatheter aortic valve implantation. *Catheterization and Cardiovascular Interventions: Official Journal of the Society for Cardiac Angiography & Interventions*.

[63] Chen S, Dizon JM, Hahn RT, Pibarot P, George I, Zhao Y (2024). Predictors and 5-Year Clinical Outcomes of Pacemaker After TAVR: Analysis From the PARTNER 2 SAPIEN 3 Registries. *JACC. Cardiovascular Interventions*.

[64] Pagnesi M, Kim WK, Baggio S, Scotti A, Barbanti M, De Marco F (2023). Incidence, Predictors, and Prognostic Impact of New Permanent Pacemaker Implantation After TAVR With Self-Expanding Valves. *JACC. Cardiovascular Interventions*.

[65] Sandau KE, Funk M, Auerbach A, Barsness GW, Blum K, Cvach M (2017). Update to Practice Standards for Electrocardiographic Monitoring in Hospital Settings: A Scientific Statement From the American Heart Association. *Circulation*.

[66] Vahanian A, Beyersdorf F, Praz F, Milojevic M, Baldus S, Bauersachs J (2022). 2021 ESC/EACTS Guidelines for the management of valvular heart disease. *European Heart Journal*.

[67] Hosseini Mohammadi NS, Tavakoli K, Taebi M, Zafari A, Riahi M, Molaei MM (2025). Comparative Prognostic Value of Risk Factors for Predicting Pacemaker Implantation After Transcatheter Aortic Valve Replacement: A Systematic Review and Network Meta-Analysis. *The American Journal of Cardiology*.

[68] Bourenane H, Galand V, Boulmier D, Leclercq C, Leurent G, Bedossa M (2021). Electrophysiological Study-Guided Permanent Pacemaker Implantation in Patients With Conduction Disturbances Following Transcatheter Aortic Valve Implantation. *The American Journal of Cardiology*.

[69] Sammour Y, Krishnaswamy A, Kumar A, Puri R, Tarakji KG, Bazarbashi N (2021). Incidence, Predictors, and Implications of Permanent Pacemaker Requirement After Transcatheter Aortic Valve Replacement. *JACC. Cardiovascular Interventions*.

[70] Alabdaljabar MS, Eleid MF (2023). Risk Factors, Management, and Avoidance of Conduction System Disease after Transcatheter Aortic Valve Replacement. *Journal of Clinical Medicine*.

[71] Aymond JD, Benn F, Williams CM, Bernard ML, Hiltbold AE, Khatib S (2021). Epidemiology, evaluation, and management of conduction disturbances after transcatheter aortic valve replacement. *Progress in Cardiovascular Diseases*.

[72] Verhemel S, Nuis RJ, van den Dorpel M, Adrichem R, de Sá Marchi MF, Hirsch A (2024). Computed tomography to predict pacemaker need after transcatheter aortic valve replacement. *Journal of Cardiovascular Computed Tomography*.

[73] Hegeman RRMJJ, van Ginkel DJ, Laengle S, Timmers L, Rensing BJWM, de Kroon TL (2024). Preoperative computed tomography-imaging with patient-specific computer simulation in transcatheter aortic valve implantation: Design and rationale of the GUIDE-TAVI trial. *American Heart Journal*.

[74] Wang J, Zhu J, Li H, Wu S, Li S, Yao Z (2025). Multimodal Visualization and Explainable Machine Learning-Driven Markers Enable Early Identification and Prognosis Prediction for Symptomatic Aortic Stenosis and Heart Failure With Preserved Ejection Fraction After Transcatheter Aortic Valve Replacement: Multicenter Cohort Study. *Journal of Medical Internet Research*.

[75] Yoon SH, Lefèvre T, Ahn JM, Perlman GY, Dvir D, Latib A (2016). Transcatheter Aortic Valve Replacement With Early- and New-Generation Devices in Bicuspid Aortic Valve Stenosis. *Journal of the American College of Cardiology*.

[76] Fraccaro C, Buja G, Tarantini G, Gasparetto V, Leoni L, Razzolini R (2011). Incidence, predictors, and outcome of conduction disorders after transcatheter self-expandable aortic valve implantation. *The American Journal of Cardiology*.

